# Doc2Hpo: a web application for efficient and accurate HPO concept curation

**DOI:** 10.1093/nar/gkz386

**Published:** 2019-05-20

**Authors:** Cong Liu, Fabricio Sampaio Peres Kury, Ziran Li, Casey Ta, Kai Wang, Chunhua Weng

**Affiliations:** 1 Department of Biomedical Informatics, Columbia University, New York, NY 10032, USA; 2 Center for Cellular and Molecular Therapeutics, Children's Hospital of Philadelphia, Philadelphia, PA 19104, USA; 3 Department of Pathology and Laboratory Medicine, University of Pennsylvania, Philadelphia, PA 19104, USA

## Abstract

We present Doc2Hpo, an interactive web application that enables interactive and efficient phenotype concept curation from clinical text with automated concept normalization using the Human Phenotype Ontology (HPO). Users can edit the HPO concepts automatically extracted by Doc2Hpo in real time, and export the extracted HPO concepts into gene prioritization tools. Our evaluation showed that Doc2Hpo significantly reduced manual effort while achieving high accuracy in HPO concept curation. Doc2Hpo is freely available at https://impact2.dbmi.columbia.edu/doc2hpo/. The source code is available at https://github.com/stormliucong/doc2hpo for local installation for protected health data.

## INTRODUCTION

Variant interpretation remains a major barrier to understanding hereditary disorders, despite the advances in sequencing technology (1). Recent studies have shown that phenotypic information helps prioritize disease-related genes and variants, and multiple computational gene-prioritization tools, such as Phenolyzer (2), Phenomizer (3), PheVor (4) and Exomiser (5), have been developed to aid the interpretation of results from clinical genome or exome sequencing. In particular, we recently demonstrated that extraction of phenotype terms from clinical texts enabled improved interpretation of sequencing data and expedited clinical diagnosis of patients with suspected Mendelian diseases (6). The Human Phenotype Ontology (HPO) (7) is one of the most popular standardized vocabularies of human phenotypic abnormalities. The latest version of HPO contains >13 000 concepts with over 156 000 annotations to human hereditary diseases. The process for clinical researchers to manually curate the standardized HPO concepts from electronic health record (EHR) narratives remains laborious. A newly available tool ‘Phenotoro’ can aid in the encoding of manually curated phenotypes with the HPO standard (8). However, the use of this tool requires extensive knowledge of HPO and depends on manual selection and insertion of HPO concepts. The process can still be error prone given the subjective nature of the process.

Several automated concept extraction tools are now available from the recent community efforts devoted to identifying clinical entities in clinical notes using natural language processing (NLP) techniques, however the accuracy of concept mapping of these tools is still unsatisfactory (9). To address the critical need for efficient and accurate HPO concept curation, we developed Doc2Hpo, a novel web application based on the human-computer collaboration design principle (10) for semi-automatically extracting standardized HPO terms from clinical text with automated concept normalization using HPO. Doc2Hpo automatically highlights clinical entities with their corresponding HPO mappings to facilitate manual review and revision if needed. Users can export the final list of HPO concepts to gene prioritization tools for further analysis.

## IMPLEMENTATION

**Figure 1.**
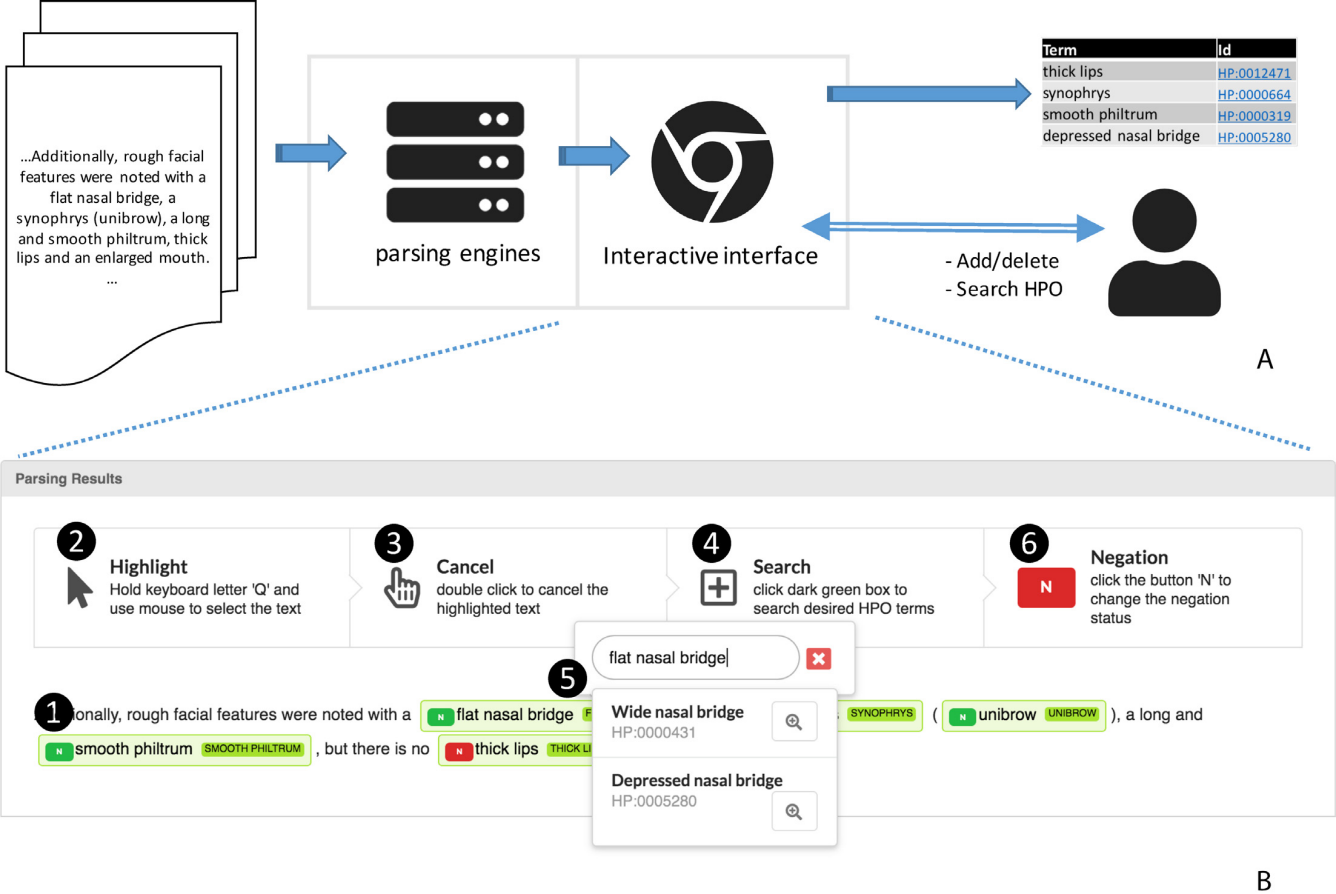
(**A**) Architecture of Doc2Hpo. (**B**) Interactive user interface. 1. Automated extraction includes three parts: light green highlights the clinical entity identified within the text, the term in the appended dark green box identifies the standardized HPO concept, and the ‘N’ button appended indicates the negation modifier (red for negation). 2–3. The user can highlight any text and add an HPO concept. The user can also double click to delete an erroneous extraction; 4–5. The user can change the standardized HPO concept by clicking the dark green box. The user could search for the desired HPO concept and make updates in real-time. 6. The user can click the ‘N’ button to change the negation modifier.

The web service is implemented in JAVA Spring Model-View-Controller (MVC) framework. The frontend annotation interface was built based on a text highlighter, mark.js (https://markjs.io/), written in JavaScript. The real-time HPO search function was implemented based on the API provided by https://hpo.jax.org/app/. The architecture of Doc2Hpo is illustrated in Figure 1A. The backend parsing engine will automatically recognize the phenotype concepts from the user input, and negation detection is available via the NegEx algorithm (11). Options such as ‘allow partial match’ are available for users to configure Doc2Hpo's behavior to tailor its performance to their needs. Users can choose one of five NLP engines for HPO concept extraction. The first engine is a string-based method leveraging the Aho–Corasick algorithm (12) for speedy concept extraction. All HPO terms and their synonyms under ‘phenotypic abnormality’ (HP:0000118) are used for the string-based search. The second engine uses a locally configured MetaMap (13) server via the Java API to extract UMLS CUI terms. MetaMap first identifies candidate clinical terms through lexical and syntactic analysis and maps them to standard UMLS concepts. The UMLS concepts are then mapped to HPO concepts following the mapping at http://purl.obolibrary.org/obo/hp.obo. The third engine employs the online NCBO Annotator (14) API for HPO concept recognition. Different options for NCBO Annotator are exposed to users via the Doc2Hpo interface to customize the parsing. For instance, users would check the ‘longest match only’ to select only the longest matching HPO term when two different HPO terms would fit. The fourth engine is MetaMap Lite (15), a fast version of MetaMap that provides near-real time named entity recognition. Though not as rigorous as MetaMap, it is much faster. The fifth option, Ensemble by Union, considers the results generated from the previous four engines and takes the union as the final result. It lowers the false negative rate by sacrificing the execution efficiency.

## INTERACTIVE USER INTERFACE

Given the complexity of biomedical NLP (16–19), particularly in named entity recognition and concept normalization, a human-computer collaborative approach is a pragmatic solution. Doc2Hpo is designed based on this principle. One of the most useful features of Doc2Hpo is the interactive user interface that allows users to easily modify the automated extraction results. Figure 1B shows a typical parsing result and the functions provided by the user interface. Users can highlight the text to add annotations missed by machine, or double click text to delete incorrectly identified phenotypes. The dictionary look-up feature, which allows users to search for relevant concepts, distinguishes Doc2Hpo from other annotation tools. Users can simply click the dark green box to search for or modify the standard HPO concepts. This function makes Doc2Hpo most suitable for the concept recognition tasks, which differs from the named entity recognition task in the way it requires the mapping from text to standardized concepts. Users can also correct the erroneously assigned negation modifiers by clicking the prepended ‘N’ button. Finally, different outputs are available in a variety of popular formats to provide compatibility with various applications, which we will illustrate in detail in the Use Cases section.

## EVALUATION

**Figure 2.**
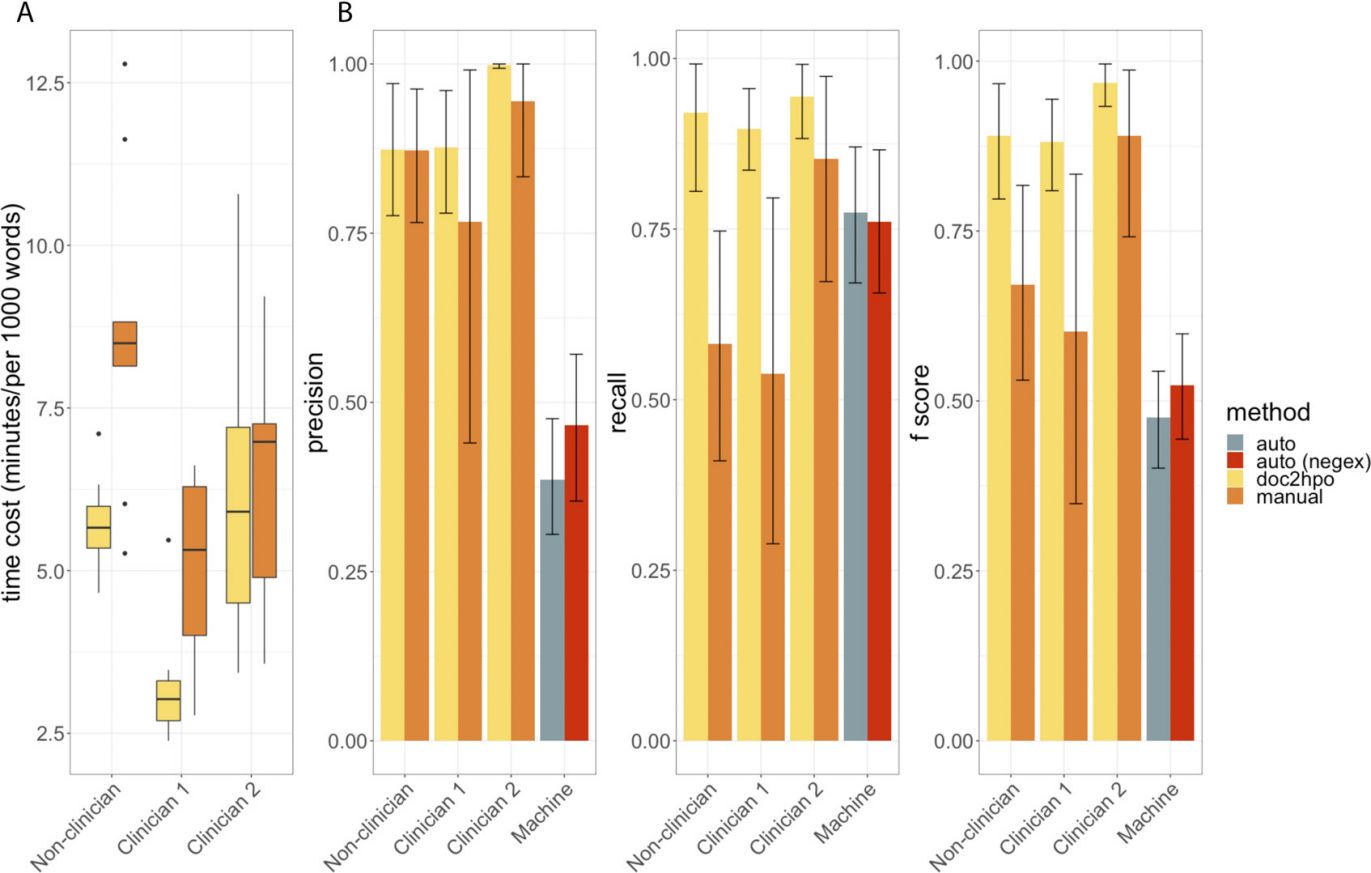
(**A**) Box and whisker plots of time consumption by using Doc2Hpo and manual curation across three different annotators. (**B**) The accuracy (precision, recall, and *F*1 score) for different approaches across different annotators. The error bars indicate the 95% confidence intervals. ‘Auto’ refers to fully automated HPO curation based on MetaMap without negation detection. ‘Auto (negex)’ refers to fully automated HPO curation based on MetaMap with negation detection. ‘Doc2hpo’ refers to Doc2Hpo-aided user curation. ‘Manual’ refers to user curation without using Doc2Hpo.

To evaluate the performance of Doc2Hpo, we compared the performance of Doc2Hpo-aided user curation (referred to as ‘Doc2Hpo’) against three other approaches: manual HPO curation, fully automated HPO curation without negation detection, and fully automated HPO curation with negation detection. Three different users (two clinicians and one researcher without clinical background) were assigned 18 clinical notes (with more than 100 distinct pairs of note-HPO concepts), obtained from NewYork-Presbyterian/Columbia University Irving Medical Center. To prevent bias, each note was randomly assigned to one or two users for manual concept curation, and then assigned to the other user(s) for Doc2Hpo-aided curation. Fully-automated HPO extraction was performed using the MetaMap parsing engine within Doc2Hpo and evaluating the resulting list of HPO terms directly (i.e. without user intervention). The gold standard was determined by consensus between the two clinicians after reviewing the combined curation results from all four approaches, resulting in 108 distinct pairs of note-HPO concepts. Both accuracy and efficiency were evaluated. Our results showed that Doc2Hpo achieved the highest accuracy and efficiency (Figure 2). Doc2Hpo achieved higher precision (0.916) than manual extraction (0.861), automated extraction (0.386), and automated extraction with negation detection (0.467). Doc2Hpo also achieved higher recall (0.921) than manual extraction (0.658), automated extraction (0.774) and automated extraction with negation detection (0.761). The average F1-score for Doc2Hpo, manual extraction, automated extraction and automated extraction with negation detection were 0.913, 0.72, 0.476 and 0.524, respectively. Compared against manual curation, Doc2Hpo-assistant manual curation made the process more efficient by reducing time consumption from ∼7 to ∼5 min per 1000 words. In addition, we found that Doc2Hpo might help reduce the knowledge gap between clinicians and non-clinical annotators by reducing the time-consumption differences. More details on evaluation design and results are described in the Supplementary Material.

## USE CASES

### Genetic councilors or physicians: automated EHR phenotype-driven gene analysis

Phenotype-driven gene analysis tools, like Phenolyzer, require physicians to input a list of HPO terms to find causal genes and variants, which requires extensive knowledge of HPO. Doc2Hpo complements these gene analysis tools by minimizing manual effort in phenotype concept curation. For instance, physicians could type or paste narrative patient descriptions, and use Doc2Hpo to identify standardized HPO concepts. They could examine and manually correct the results (if necessary) to guarantee parsing quality. By clicking the ‘Phenolyzer’ link in the ‘Physicians’ section, a Phenolyzer-compatible phenotype list will be generated in the clipboard and the users will be directed to Phenolyzer, where the standardized phenotypes can be pasted into the input box for further gene prioritization analysis.

### Phenotype data curator: standardized phenotypes collection

Clinical data curators need to identify and coordinate the flow of phenotypic data from EHR into clinical study databases. Phenotype concept extraction based on manual chart review can be laborious, inaccurate, incomplete, and inconsistent from person to person. Data curators can employ Doc2Hpo in this use case to facilitate phenotype extraction. After the Doc2Hpo parsing engines extract the initial set of recommended phenotypes, data curators can customize the results using the interactive user interface to easily add, remove, or replace current concepts. Finally, they could download the resulting collection of standardized phenotypes in Excel or PDF format.

### Annotators: NLP-assisted textual annotation

Doc2Hpo could serve as an annotation platform to enhance the efficiency of creating training corpora for standardized clinical concept recognition. A human annotator could attach a free-text training sample and annotate it using the interactive user interface. The automated annotated results could minimize annotation workload and provide guidance, while the dictionary lookup utility could improve efficiency and reduce errors. The final annotated results could be downloaded as a JSON file, which is a stand-off annotation file that identifies the start and end positions in the text it applies to with concrete standardized HPO concepts recognized.

## DISCUSSION

We present a semi-automatic tool to enable interactive, efficient and accurate HPO term curation from clinical narratives with minimal human effort. To the best of our knowledge, this is the first web application to enable text annotation with HPO using a real-time vocabulary look-up function. Additionally, we made the source code of the web application available, so that users can run a local installation of Doc2Hpo behind institutional firewalls to annotate clinical texts with protected health information. Our main contribution is this novel platform where machine and human could work collaboratively to improve the efficiency and accuracy for standardizing HPO concept recognition. Doc2Hpo outperforms fully automated extraction approaches in precision by allowing manual detection and removal of hypothetic phenotypes, terms not related to patients, or educational text. It is also better than the manual approach because the real-time dictionary look-up utility facilitates finding more standardized terms. Furthermore, by using Doc2Hpo, non-clinical annotators could achieve a similar performance as clinicians, which indicated the potential of using Doc2Hpo to reduce the cost of data curation for clinical studies. However, due to the time and effort required to produce the gold standard, the evaluation of this study is relatively small, and large-scale evaluations are wanted to confirm the findings.

Doc2Hpo provides multiple options for parsing engines, and different parsing engines tend to have different tradeoffs between accuracy and speed. In general, the ensemble parsing engine can detect more HPO concepts than the individual ones, but takes the most time to complete. Among the individual parsing engines, MetaMap can recognize more concepts but costs more processing time. By turning on negation detection, we can reduce the false positives if the user only wants the non-negated phenotypes. Based on our experience, MetaMapLite and NCBO Annotator achieve the best balance between efficiency and accuracy.

As we showed in our evaluation, it is still challenging for current NLP systems to automatically extract a comprehensive set of phenotype terms from clinical narratives. Particular difficulties for automated parsers include recognizing spelling variations (‘renal hypertension’ – ‘renovascular hypertension’), synonymy (‘double kidney’ – ‘extra kidney’), and hyponymy/hypernymy (‘skin depigmentation’ – ‘depigmentation/hyperpigmentation of skin’). By allowing users to insert new annotations for these difficult cases that existing automated parsers cannot handle well, Doc2Hpo provides means to improve the recall rate.

Other work closely related to Doc2Hpo lie in two categories, automated concept recognition and manual text annotation for NLP tasks. The NCBO Annotator (https://bioportal.bioontology.org/annotator) is a popular tool to get annotations for biomedical text with classes from the ontologies (14). Users could input free-text and select the desired ontologies, and the system will return the corresponding recognized concepts. Our work differs from NCBO Annotator in three aspects. First, as reported in recent works (9,20,21), the accuracy of machine-only based concept recognition is still low. To ensure high quality results, our interactive user interface provides several easy-to-use functions enabling users to modify the extraction results if necessary. This interactive user interface is the most important feature that distinguishes Doc2Hpo from other online concept recognition tools, including Text Annotator developed by the Monarch Initiative (https://monarchinitiative.org/annotate/text) (22). Second, multiple parsing engines were integrated in the Doc2Hpo web application, allowing users to select the appropriate parsing engine that fits their needs. Third, by implementing a negation detector, Doc2Hpo excludes negated mentions of other phenotypes and retains only patient-specific phenotypes. Finally, as a bridge to connect clinical notes and gene prioritization tools, Doc2Hpo provides multiple output formatting options to facilitate entry for different gene prioritization tools.

Different from other popular text annotation platforms, such as BRAT (http://brat.nlplab.org/) (23), ANAFORA (https://github.com/weitechen/anafora) (24), which mainly focus on named entity recognition and relation extraction, Doc2Hpo is designed specifically to annotate text for phenotype concept recognition. By using the dictionary lookup function, it could increase human productivity. In addition, although human judgement could not be replaced completely by automated annotation, our evaluation does show the recall of the annotation could be improved by using a first-round automated parsing result.

The Doc2Hpo server undergoes continuous improvement. In the future, we will incorporate more parsing engines, such as cTakes (25) and ClinPhen (26), to provide a more comprehensive ‘ensemble’ parsing results for further annotation. Doc2Hpo can also be easily extended for other standard phenotype or disease concept recognition ontologies such as OMIM (27) and Orphanet (28) to fulfil the fast expansion of phenotype related ontologies provided by the Monarch Initiative (22).

## DATA AVAILABILITY

The original clinical notes used for the current study are available from the corresponding authors on reasonable requests and institutional approvals.

## Supplementary Material

gkz386_Supplemental_Files
