# Supplementary material for: Doc2Hpo: a web application for efficient and accurate HPO concept curation
Source: Nucleic Acids Res. 2019 May 20;47(W1):W566–70. doi: 10.1093/nar/gkz386 (PMC6602487; doi:10.1093/nar/gkz386)
Supplement: gkz386_Supplemental_Files [file gkz386_supplemental_files.docx]

**Supplementary Material**

Evaluation Design

To evaluate the performance of Doc2Hpo, we compared the performance of Doc2Hpo-aided user curation (simply referred to as ‘Doc2Hpo’) against two other approaches: manual curation and fully automatic HPO extraction. Three annotators were asked to complete 18 clinical notes provided by New York-Presbyterian/Columbia University Irving Medical Center. To make the results comparable, each note was annotated by all three annotators, with one or two annotators performing manual annotation, and the remaining annotators using Doc2Hpo for assisted curation. Each annotator annotated each clinical note once: 9 notes by manual annotation, and the other 9 notes by Doc2Hpo based annotation. To avoid any potential bias introduced by batch effect, the order of documents was shuffled (i.e., the order of methods was shuffled) for annotation. Fully automated HPO extraction was performed by using the MetaMap parsing engine with default settings within Doc2Hpo and evaluating the resulting list of HPO terms directly (i.e., without user intervention).

For each note, annotators were asked to extract only patient-specific HPO terms while excluding HPO terms not directly related to the patient (e.g., family history, educational material, etc.). We also required annotators to remove negated HPO occurrences (e.g., “No prior history of hypertension.”) so that only positive HPO findings were retained. To evaluate efficiency, we measured the elapsed time for reviewing each note. The timer started when opening the file and stopped when the annotator finished entering all HPO terms for the note. During manual annotation, annotators used <https://hpo.jax.org/app/> as a dictionary to search the HPO terms. For Doc2Hpo, annotators used the string match based parsing engine with default settings to parse the note first, and then annotated the parsing results. Since the time cost varies largely due to the length of each document, we normalized the time cost by dividing by the length of each note (i.e., word count).

The ideal way to establish a gold standard is to ask another independent annotator to curate the gold standard independently for each note. However due to the small scale of this study, we decided to generate a gold standard for each note based upon consensus of the two clinicians after reviewing the combined curation results from all three approaches. The two clinical annotators discussed and generated the gold standards after all the experiments were finished so that this process would not affect their performance during annotation experiments. The final recall and precision were calculated for each note by comparing the HPO terms extracted using each approach with the gold standard.

Evaluation Results

Due to the hierarchical nature of the HPO vocabulary, we expanded the curated list and gold standard by considered all ancestors. We only considered HPO terms under the branch starting from Phenotypic abnormality (HP:0000118) (not included). We then compared each curated list against the gold standard for each note. True positive (TP) was defined as the unique number of correctly extracted HPO terms (including expanded terms). False positive (FP) was defined as the unique number of wrongly extracted HPO terms (including expanded terms). False negative (FN) was defined as the number HPO terms missed (including expanded terms). The precision, recall and F1-score were defined as following:

$$Precision=\frac{TP}{TP+FP}$$

$$Recall=\frac{TP}{TP+FN}$$

$$F1=2*\frac{precision*recall}{precison+recall}$$

We then calculated a confidence interval for the precision and recall using bootstrapping. For each of 1000 trials, 18 notes were sampled with replacement to generate the distribution of the precision and recall. The 95% confidence interval was then calculated as to be between the 2.5th and the 97.5th percentiles.
